# Tattooing following snake envenomation

**DOI:** 10.11604/pamj.2025.52.80.48675

**Published:** 2025-10-21

**Authors:** Amit Toshniwal, Varun Hanumanthaiah

**Affiliations:** 1 Department of Respiratory Medicine, Datta Meghe Institute of Higher Education and Research, Wardha, Maharashtra, India,; 2 Department of Dermatology, Venerology and Leprosy, Datta Meghe Institute of Higher Education and Research, Wardha, Maharashtra, India

**Keywords:** Snake bite, zoonotic disease, toxicology

## Image in medicine

A 32-year-old female from rural India presented to the emergency department with a history of a snake bite that had occurred two hours earlier on the left foot. Upon examination, a small bite mark was visible, and the surrounding area was swollen and warm to the touch. However, there were no neurological abnormalities observed in the patient. Initially, the diagnosis was nontoxic snake envenomation, and conservative management was initiated, and the patient was closely monitored. After three days, the patient returned to the emergency department with blackish discoloration over the left foot. Upon examination, the area was no longer warm to the touch and was not tender. The patient had been counseled about post-envenomation tattooing and was asked to follow up. This case highlights the unusual presentation of tattooing after snake envenomation and emphasizes the importance of thorough counseling in such situations.

**Figure 1:**
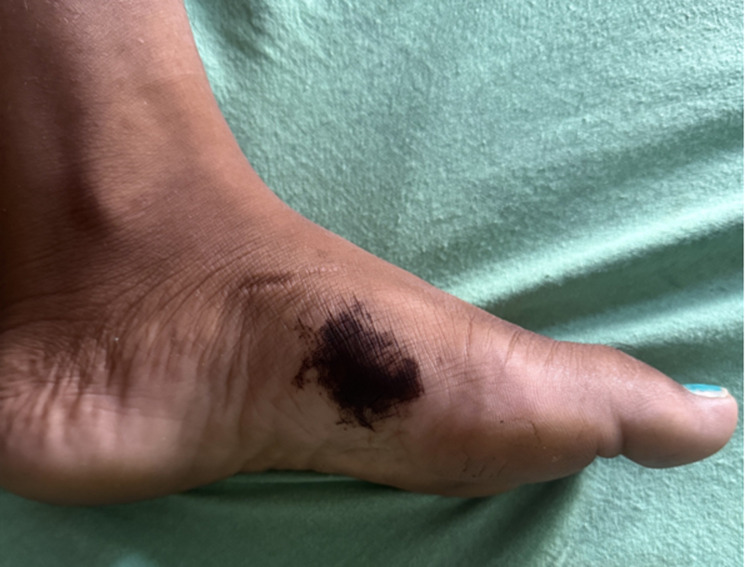
tattooing over the left foot after a snakebite

